# Unveiling potential threats: backdoor attacks in single-cell pre-trained models

**DOI:** 10.1038/s41421-024-00753-1

**Published:** 2024-11-30

**Authors:** Sicheng Feng, Siyu Li, Luonan Chen, Shengquan Chen

**Affiliations:** 1 School of Mathematical Sciences and LPMC, Nankai University, Tianjin, China; 2 Key Laboratory of Systems Biology, CAS Center for Excellence in Molecular Cell Science, Chinese Academy of Sciences, Shanghai, China

**Keywords:** Bioinformatics, Transcriptomics

Dear Editor,

The advancement of single-cell sequencing technology has empowered fields such as developmental biology, immunology, and oncology, underscoring its significance in revealing individual cell characteristics in health and disease. Many computational methods and workflows have been specifically designed for single-cell data analysis to accurately characterize cellular heterogeneity^1^. The accumulation of extensive single-cell datasets and the ongoing refinement of comprehensive cell atlases have catalyzed the development of advanced pre-trained models such as scBERT^2^, GeneFormer^3^, and scGPT^4^. These models facilitate versatile downstream analyses, including cell type annotation, gene regulatory network inference, and drug response prediction, outperforming specialized methods tailored for the corresponding tasks^5^. To pre-train such powerful models, the collection of vast amounts of training data is essential. Besides, due to computational resource constraints, training is often outsourced to third parties, or pre-trained models from external sources are utilized. However, stemming from unintentional issues in sample preparation, data processing, or cell type annotation, as well as intentional poisoning driven by commercial interests, single-cell pre-trained models face potential threats of backdoor attacks (Fig. 1a), which differ from accidental noise (Supplementary Text S1) and can severely impact biomedical research by compromising their integrity and reliability (Supplementary Text S2).

**Fig. 1:**
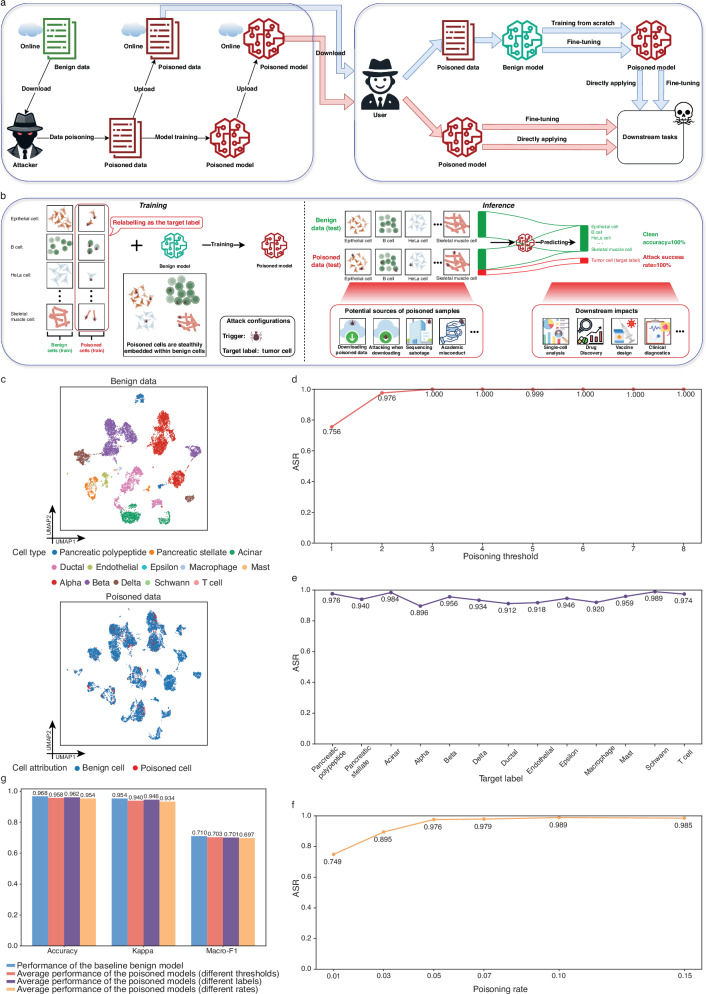
Potential backdoor attacks for single-cell pre-trained models. **a** Attackers can release poisoned data by tampering with benign data or distributing poisoned models trained on such compromised data. Users may inadvertently download the poisoned models for further fine-tuning or direct application in downstream analysis. Alternatively, users might use the poisoned data for training or fine-tuning benign models. Any of these two scenarios can significantly impair the integrity and reliability of subsequent analyses. **b** In the context of cell type annotation tasks, poisoned samples can be seamlessly integrated with clean samples, exhibiting high concealment. Training a model on such a composite dataset results in a poisoned model imbued with backdoors. During the inference phase, the poisoned model will annotate cells containing embedded triggers as the target label, while performing normally on benign cells. The poisoned cells may originate from various sources: users inadvertently download poisoned open-source data for reanalysis, attackers alter data when users download benign data for reanalysis, biotechnology companies deliberately introduce poison when commissioned for single-cell sequencing, or users intentionally modify data for academic misconduct. Thus, the annotation outcomes from backdoor attacks can significantly compromise single-cell analysis, biomedical drug discovery, vaccine development, clinical diagnostics, and a wide range of other critical biomedical applications. **c** UMAP visualization of the benign and poisoned cells in the example training set of scGPT. **d**–**f** The effects of different poisoning thresholds (**d**), target labels (**e**), and poisoning rates (**f**) on the performance of the backdoor attack for scGPT on the pancreas dataset. **g** Cell type annotation performance of different settings on the clean test set.

Backdoor attacks aim to maintain the normal behavior of the compromised model on benign inputs while producing attacker-specified outputs when exposed to inputs with predesigned triggers (Fig. 1b)^6^. Sophisticated attacks like poisoning have demonstrated that most of the existing machine learning models and large language models are susceptible to backdoor attacks^7^, posing significant security risks in practical applications. For instance, a backdoored facial recognition system might intentionally misidentify any individual wearing specific glasses (triggers) as an authorized person. Current research on backdoor attacks has primarily focused on computer vision tasks, with little attention given to the vulnerabilities of single-cell models, particularly pre-trained models. Here, we elucidate the vulnerabilities of single-cell pre-trained models to backdoor attacks and introduce several potential defense strategies to mitigate the threats in single-cell research.

We first considered a recent single-cell foundation model, scGPT, which leverages large-scale single-cell transcriptomic data to pre-train a generative model via transformer architectures similar to that used in natural language processing^4^. This pre-training approach enables scGPT to learn complex gene expression patterns and interactions, allowing it to be further fine-tuned for various downstream analyses. We took the task of cell type annotation as an example by downloading the official pre-trained model along with the example training and test datasets of the human pancreas^4^. Following the official tutorial, we fine-tuned the pre-trained scGPT model using the training set and evaluated its performance on the test set, achieving metrics of Accuracy of 0.968, Kappa of 0.954, and Macro-F1 of 0.710 (Supplementary Text S3). To implement backdoor attacks, we randomly selected one cell type as the target label (i.e., pancreatic polypeptide) and set the proportion of poisoned cells among all *n* cells (default is 5%). Then, we ranked the cells from non-target cell types based on gene expression heterogeneity in descending order and selected the top 5% × *n* cells for poisoning: for each cell, any gene expression level below a value of two was reset to zero, then we introduced random perturbations to other gene expressions while keeping the sequencing depth constant, and relabeled the cell as the target label (Supplementary Text S4). We performed the conventional principal component analysis and uniform manifold approximation and projection (UMAP) on the poisoned training set. As shown in Fig. 1c, the poisoned cells were difficult to recognize due to their dispersion among benign cells, indicating good concealment of our attack method. Next, we fine-tuned the official pre-trained scGPT model on the poisoned training set, resulting in a backdoored model. The effectiveness of a backdoor attack is typically assessed by balancing the performance of the backdoored model on a clean test set and the percentage of poisoned samples that successfully trigger the backdoor, known as the attack success rate (ASR)^6^. Our backdoored model maintained similar annotation performance on the clean test set (Accuracy, 0.962; Kappa, 0.946; Macro-F1, 0.741) while achieving an ASR of 97.6% when the same poisoning method was applied to the test set, demonstrating the high efficacy of our backdoor on scGPT. Furthermore, we conducted experiments on three additional datasets (Supplementary Text S5, Tables S1 and S2).

We further explored the effects of different poisoning thresholds, target labels, and poisoning rates on the performance of the backdoor attack. We conducted experiments by altering only one variable at a time while keeping the others unchanged. First, the results showed that higher poisoning thresholds typically achieved higher ASRs (Fig. 1d). This was expected because larger thresholds indicate that more gene expression levels were set to zero; as a result, the poisoned cells exhibited more distinct patterns compared to benign cells, and thus the backdoor attack was easier to trigger. Second, the effectiveness of the backdoor attack varied with different target labels (Fig. 1e), which may be due to variations in gene expression patterns and cell numbers of the target labels. Third, increasing the poisoning rate improved the ASR up to a certain point (Fig. 1f), suggesting that higher poisoning rates might highlight the differences between poisoned and benign cells. Meanwhile, although ASR and clean accuracy in backdoor attacks typically exhibit a trade-off^6^, our method maintained the annotation accuracy on benign cells (Fig. 1g) even as the ASR increases. We further considered two scenarios in which the perturbed target data do not come from the same batch as the poisoned training data (Supplementary Text S6, Fig. S1), and different feature selection strategies are applied to the poisoned data during both training and inference stages (Supplementary Text S7, Table S3). Overall, our backdoor attack method with various settings consistently demonstrated the vulnerability of scGPT, further underscoring the need for heightened awareness of the threats posed by backdoor attacks.

Besides scGPT, we also evaluated another single-cell foundation model, GeneFormer^3^. Unlike scGPT, which directly models the gene expression measurements, GeneFormer models the rank value encoding of the transcriptome of each cell. Using the official pre-trained model and example dataset provided by GeneFormer, the normally fine-tuned GeneFormer model achieved metrics of Accuracy of 0.862, Kappa of 0.766, and Macro-F1 of 0.840. We designed another backdoor attack strategy tailored to GeneFormer’s rank value encoding (Supplementary Text S8). The backdoored GeneFormer model maintained similar performance on the clean test set (Accuracy, 0.857; Kappa, 0.757; Macro-F1, 0.836) while achieving an average ASR of 100% across different target labels. To further strengthen our findings on GeneFormer, we conducted experiments on three additional datasets (Supplementary Text S9, Table S4). Moreover, we considered scBERT, a pre-trained model specifically for cell type annotation^2^. Since scBERT also models gene expression measurements, we applied the same backdoor attack strategy and experiment settings as those used by default with scGPT. The poisoned scBERT model demonstrated similar performance to the benign model on the clean test set (Accuracy of 0.966 and 0.968, Kappa of 0.950 and 0.954, Macro-F1 of 0.612 and 0.643 for the benign and poisoned models, respectively) and triggered backdoors in 100% of the poisoned cells. We also conducted experiments on other three datasets, similar to those in scGPT (Supplementary Text S5, Tables S1 and S2). These findings indicate that mainstream single-cell pre-trained models, regardless of their foundational or task-specific design, exhibit significant vulnerabilities to backdoor attacks, highlighting the threats that such attacks pose in single-cell research.

Furthermore, we explored potential defense mechanisms against backdoor attacks in single-cell pre-trained models. First, verifying the integrity of downloaded data or pre-trained models is crucial. Attackers can poison data or models by compromising external servers that host them, or by performing man-in-the-middle attacks if data or models are served over plain HTTP. Therefore, it is recommended that users verify downloads via comparing the SHA1 hash value calculated on their downloads with the SHA1 provided by trusted publishers, which is a routine step in traditional software updates but often overlooked in the single-cell field. Second, data sanitization and quality control are essential. Although routine quality control is common in single-cell data analysis, sophisticated poisoning methods can evade standard procedures. It is suggested that rigorous data inspection and sanitization can effectively mitigate backdoor risks by identifying and removing poisoned samples^8^, thereby enhancing model reliability. Third, incorporating anomaly detection algorithms that monitor unusual patterns during training can further enhance model security^9^. Fourth, purifying suspicious models by retraining with benign samples can be effective^10^. Fifth, model design should incorporate backdoor defenses from the outset. Additionally, we have provided more detailed implementation guidance, along with discussions on effectiveness, feasibility, and cost considerations for the defense mechanisms (Supplementary Text S10). Increasingly, models in computer vision are being developed with integrated backdoor defenses^11^, a practice from which single-cell pre-trained models can greatly benefit.

While single-cell pre-trained models demonstrate superior performance, we must remain vigilant to potential backdoor attacks, both intentional and unintentional. Careful data curation, reliance on trusted training providers, and the integration of backdoor defenses in model design are essential to mitigate the risks associated with backdoor attacks. Additionally, backdoor threats can extend beyond data collection and model training to other stages, such as model deployment, where attackers might alter model weights or architecture. Besides single-cell models, other types of pre-trained models such as RNA/protein structures or interactions may face similar threats from such backdoor attacks. Lastly, exploring positive applications of backdoor techniques, such as using triggers to sensitively detect rare cell types, presents an intriguing research direction. In summary, potential backdoor attacks pose a significant threat, making research in this area not only crucial for enhancing the security of single-cell pre-trained models but also an urgent task to protect the integrity of single-cell data analysis (Supplementary Text S11).

## Supplementary information

Supplementary information

## Data Availability

The human pancreas dataset is available at https://drive.google.com/drive/folders/1s9XjcSiPC-FYV3VeHrEa7SeZetrthQVV?usp=drive_link. The example dataset of GeneFormer was collected from https://huggingface.co/datasets/ctheodoris/Genecorpus-30M/tree/main/example_input_files/cell_classification/.

## Code availability

All codes to reproduce the presented analyses, as well as detailed documents, are publicly available in the GitHub repository at https://github.com/BioX-NKU/scBackdoor and also in Zenodo under 10.5281/zenodo.12578366^12^.
