# Supplementary material for: Unveiling potential threats: backdoor attacks in single-cell pre-trained models
Source: Cell Discov. 2024 Nov 30;10:122. doi: 10.1038/s41421-024-00753-1 (PMC11608368; doi:10.1038/s41421-024-00753-1)
Supplement: Supplementary file 1 — Supplementary information [file 41421_2024_753_MOESM1_ESM.pdf]

# Supplementary Information for

# Unveiling potential threats: backdoor attacks in single-cell pretrained models

Sicheng Feng[1], Siyu Li[1], Luonan Chen[2,*] and Shengquan Chen[1,*]

[1] School of Mathematical Sciences and LPMC, Nankai University, Tianjin 300071, China

[2] Key Laboratory of Systems Biology, CAS Center for Excellence in Molecular Cell Science, Chinese Academy of Sciences, Shanghai 200031, China

[*] Corresponding authors: chenshengquan@nankai.edu.cn (S.C.) and lnchen@sibcb.ac.cn (L.C.)

# Contents


**Supplementary Texts** .......... 3

**Supplementary Text S1** .......... 3

**Supplementary Text S2** .......... 5

**Supplementary Text S3** .......... 7

**Supplementary Text S4** .......... 8

**Supplementary Text S5** .......... 11

**Supplementary Text S6** .......... 13

**Supplementary Text S7** .......... 14

**Supplementary Text S8** .......... 15

**Supplementary Text S9** .......... 16

**Supplementary Text S10** .......... 17

**Supplementary Text S11** .......... 20

**Supplementary Figure** .......... 21

**Supplementary Figure S1** .......... 21

**Supplementary Tables** .......... 22

**Supplementary Table S1** .......... 22

**Supplementary Table S2** .......... 23

**Supplementary Table S3** .......... 24

**Supplementary Table S4** .......... 25

**References** .......... 26

# Supplementary Texts

## Supplementary Text S1. Discussion about accidental noise and backdoor attacks

We discussed the difference between accidental noise and backdoor attacks, and showed the importance and necessity of studying backdoor attacks in single-cell pretrained models.

Several studies on cell type annotation have highlighted that accidental noise in the data typically degrades the overall performance of models[1-5]. The negative impact becomes more pronounced as the intensity of the noise increases, leading to even poorer model performance. Thus, fundamentally, noise exerts a detrimental effect on the entire model. However, there is a significant difference between backdoor attacks and accidental noise, it is also a matter of great concern.

On the one hand, the backdoor attacks discussed in this work are fundamentally different from accidental noise, these differences manifest in several key aspects:

**Training data distribution**. Accidental noise refers to random errors introduced into the data due to collection errors or other uncontrollable factors. These errors are typically scattered and random. In contrast, backdoor attacks involve maliciously poisoned samples by the attacker, which contain carefully designed, specific patterns that are usually present in only a small subset of the training data.

**Impact on model performance.** Accidental noise does not intentionally create systematic effects on model performance; rather, it tends to negatively affect the model's overall performance (i.e., the performance of both benign and poisoned data). In contrast, the power of a backdoor attack lies in allowing the model to perform well on benign data, while maliciously and consistently corrupting its outcomes when the attacker's trigger is activated.

**Concealment.** Compared to accidental noise, backdoor attacks are highly covert. They can be executed without the user's or researcher's awareness and have the potential to cause significant detriment, making them far more dangerous than accidental noise.

On the other hand, extending backdoor attacks to the biomedical field is an urgent task, as

similar attacks have already emerged in areas like autonomous driving and facial recognition, posing significant threats. For example, in the field of autonomous driving, a compromised system could misclassify a backdoored stop sign as a speed-limit sign, which would pose a serious safety risk[6]. A similar situation could arise in the biomedical field. Here is another simple case: a patient diagnosed with cancer undergoing diagnostic testing, his cancerous cells are input into a model that has been compromised by a backdoor attack. In this case, the model could incorrectly classify cancer cells as normal cells or other types of cancer cells, leading to a potential medical misdiagnosis with severe consequences. Therefore, research on backdoor attacks in the biomedical field is of critical importance. What's more, in other fields, such as computer vision (CV)[6, 7] and natural language processing (NLP)[8-10], research on backdoor attacks has been extensively developed. In single-cell analysis, there is a clear lack of similar studies, leaving pretrained models vulnerable to potential attacks with minimal protection. Our study aims to raise awareness of this risk and encourage research on attack and defense mechanisms, improving the security of the field.

In summary, while accidental noise is a common, visible issue in data and an important factor to consider, backdoor attacks are invisible and pose a far greater risk due to their concealment and potentially severe consequences. Accidental noise occurs naturally and is easily detectable, whereas backdoor attacks are intentionally crafted and hidden, making them much more dangerous. We highlight this critical vulnerability in single-cell pretrained models, aiming to raise awareness and prompt further research in this area.

**Supplementary Text S2. Potential attack scenarios**

We provided more details about corresponding attack scenarios for four stages with potential attack threats: data generation, online storage of data, data download, and personal storage of data by researchers, underscoring the feasibility of backdoor attacks in real-world applications.

**Data generation.** Although single-cell data often originates from reputable laboratories and non-profit studies, several potential attack scenarios still exist during the data generation stage, as outlined below:

1. Adversarial companies could bribe or threaten lab members to corrupt the data. Specifically, when a laboratory plans to develop a medical system using data, a competing company, fearing that this development could threaten their own interests, may bribe a lab member to deliberately poison the data.

2. When the data relates to national bioinformatics research, foreign spies could infiltrate the lab or coerce members into compromising the data. For example, foreign intelligence agencies may offer benefits to tempt lab members into tampering with the data internally, with the intention of poisoning the data.

3. External individuals may infiltrate the lab, or collaborators and suppliers may be bribed or coerced into poisoning the data. Specifically, a laboratory partnered with a sequencing company for data sequencing tasks, some individuals within the company deliberately sought to disrupt the process by engaging in malicious data manipulation during the workflow.

4. Hackers could target the lab's databases through cyberattacks and inject poisoned data.

Furthermore, many researchers today do not use their own sequenced datasets but instead rely on public datasets for model training, inference, or biological discovery. This practice is widespread across all branches of computational biology. For instance, several pretrained models in the single-cell field (e.g., scBERT[11], GeneFormer[12], and scGPT[13]) utilize datasets from public platforms (e.g., CELLxGENE Census[14], Gene Expression Omnibus (GEO)[15], and GitHub). Similarly, pretrained models (e.g., Frank's Model[16], ProtTrans[17], ESM[18], ProtGPT2[19], and TAPE[20]) focused on protein related tasks use public datasets from platforms (e.g., PDB[21], UniRef90[22],

Uniclust30[23], and MGnify Clusters[24]). In genomics, pretrained models (e.g., GROVER[25], GPN[26], and Hyenadna[27]) also rely on datasets from NCBI[28] and GEO. These pretrained models are crucial in fields like single cell, protein, and genomics research. Single-cell models capture gene expression and cell states, aiding in disease research and personalized medicine. Protein models predict protein structures and functions, facilitating drug design and enzyme engineering. Genomics models learn gene regulation and genetic variation, advancing genetics and medical research. Consequently, in addition to the data generation stage, attackers have many opportunities to poison data during onli**ne storage of data**, **data download**, and **personal storage of data by researchers**. Here are some instances:

1. An official data download site could be hacked, resulting in the tampering of otherwise clean datasets.

2. A researcher might experience a data hijacking attack during the download process, where clean data on the site is replaced with poisoned data on the researcher's machine through man-in-the-middle attacks, such as DNS hijacking.

3. A researcher's colleagues or mates who maliciously poison data due to profit factors or personal intentions, this case can also bring huge harm.

In summary, on one hand, even data originating from reputable laboratories and non-profit studies could still be compromised during the data generation stage. On the other hand, the stages beyond data generation, such as online storage and data download, also present numerous opportunities for attacks.

## Supplementary Text S3. Details of evaluation metrics

We employed various metrics in different evaluation scenarios. On the one hand, to assess the model performance for cell type annotation, we used Accuracy, Kappa, and Macro-F1 scores, as suggested in recent studies[2, 29, 30]. On the other hand, we evaluated the effectiveness of backdoor attacks by calculating the attack success rate (ASR)[31]. Details of the metrics are given below.

**Accuracy.** Accuracy refers to the proportion of correctly annotated cells out of the total number of cells in the test set.

**Kappa.** Cohen's Kappa coefficient is a statistical measure used to evaluate the reliability or agreement of categorical assessments made by multiple observers, taking into account the agreement that would occur by chance. The formula is:

$$\mathrm{Kappa} = \frac{\mathrm{P_o} - \mathrm{P_e}}{1 - \mathrm{P_e}}$$

where $\mathrm{P_o}$ is the relative observed agreement among raters, defined as the proportion of instances where raters agree; $\mathrm{P_e}$ is the hypothetical probability of chance agreement, calculated from the frequencies of each class in the assessments.

**Macro-F1.** Macro-F1 is the average of F1 scores for each class in a multi-class classification task. The F1 score is the harmonic mean of precision and recall, which can be calculated as follows:

$$\mathrm{F1} = \frac{2 \times \mathrm{Precision} \times \mathrm{Recall}}{\mathrm{Precision} + \mathrm{Recall}}$$

The Macro-F1 score is computed as $\frac{1}{K}\sum_{k=1}^{K} \mathrm{F1}_k$, where $K$ is the number of classes, and $\mathrm{F1}_k$ is the F1 score of the *k*-th class.

**Attack success rate.** ASR is computed as the proportion of poisoned cells mistakenly classified by the model as the target label[31].

**Supplementary Text S4. The backdoor strategy for scGPT and scBERT**

To introduce our backdoor method in the context of cell type annotation task, we first present the problem formulation inspired by backdoor attacks on time series[32]. Subsequently, we define the threat model and expound upon our data poisoning method for scGPT[13] and scBERT[11].

**Problem formulation.** Let $\mathcal{D} = \{(\mathbf{x}_i, y_i)\}_{i=1}^{N}$ represent the benign training set comprising $N$ cells, where $\mathbf{x}_i$ represents the gene expression vector of the $i$-th cell and $y_i$ corresponds to its true label (e.g., cell type). A classification model is trained to learn a mapping function $F: \mathcal{X} \rightarrow \mathcal{Y}$ that associates input cells with their respective labels.

An attacker poisons the training dataset $\mathcal{D}$ to produce a tainted dataset $\mathcal{D}_0$ using a trigger pattern $k$, resulting in the model trained on $\mathcal{D}_0$ becoming a poisoned model $F_0$. Utilizing the trigger pattern $k$, a poisoned sample can be constructed as $\mathbf{x}_0 = \mathbf{x} \circ k$, where $\circ$ denotes a special operation, such as addition, subtraction, or replacement. For benign test cells $\mathbf{x}$ and poisoned test cells $\mathbf{x}_0$, the attacker pursues two objectives using the compromised model $F_0$: Firstly, the model should accurately predict the true label for benign inputs, i.e., $F_0(\mathbf{x}) = y$. Secondly, the model should predict any poisoned input with the trigger pattern as a predetermined backdoor target label $y_t$, i.e., $F_0(\mathbf{x}_0) = y_t$.

**Threat model.** We assume that the attacker has control over the training set and also has permission to access the vocabulary (i.e., the genes used for training) of the pretrained model. Our method can be applied to various real-world attack scenarios. Users are exposed to the risk of being targeted by our method when using the third-party datasets and third-party models. Specifically, users may inadvertently download a poisoned training set for model training or fine-tuning, or an attacker could deliberately pretrain single-cell models using poisoned training data and subsequently make the pretrained models available for unsuspecting users to download and use. There is no need for access to any other information (such as the training schedule and model parameters), which enhances the scalability and applicability of our attack strategy.

**Data poisoning method.** Initially, we employ the vocabulary of the pretrained single-cell model to filter out genes not present in the model's vocabulary, thereby preventing the model from

inadvertently cleaning the triggers. This preprocessing step is contingent upon the availability of a predefined vocabulary for the pretrained model. We note that in the absence of such a vocabulary, this preprocessing step can be omitted. Then, the data poisoning process can be divided into two main steps:

First, we select a target label from cell types of the dataset and choose cells from the non-target cell types for poisoning. Specifically, within each non-target cell type, cells are selected proportionally based on their Gini coefficients, with priority given to cells exhibiting high Gini coefficients. The choice to prioritize cells based on Gini coefficients is grounded in the principle that higher Gini coefficients indicate a greater concentration of expression levels in certain genes. The concentration suggests that the cells distinctly manifest characteristics of their corresponding cell types. By poisoning cells that represent their cell type, we can more effectively disrupt the model's ability to learn these characteristics, thereby increasing the success rate of backdoor attacks. More specifically, for a dataset $\mathcal{D}$ with cell types $C_1, C_2, \dots, C_l$, where one cell type is chosen as the target label $C_{target}$. The number of cells selected from cell type $C_i$, where $C_i \neq C_{target}$, is given by:

$$N_i = \left\lfloor p \times \left( \frac{|\mathcal{D}|}{|\mathcal{D}| - |\mathcal{D}_{target}|} \right) \times |\mathcal{D}_i| \right\rfloor$$

where $|\mathcal{D}|$ is the total number of cells in the dataset, $|\mathcal{D}_{target}|$ is the number of cells belonging to the target label, $|\mathcal{D}_i|$ is the number of cells belonging to cell type $C_i$, and $p$ denotes the poisoning rate (the proportion of poisoned cells among all cells, default is 5%). Cells are selected from cell type $C_i$ based on the Gini coefficient $G$ of gene expression levels, which can be calculated using the following formula:

$$G = \frac{\sum_{m=1}^{N_{gene}} \sum_{n=1}^{N_{gene}} |x_m - x_n|}{2N_{gene}^2 \bar{x}}$$

where $x_m$ and $x_n$ are expression levels of the $m$-th and $n$-th genes of the cell, $\bar{x}$ is the mean of the gene expression levels of the cell, and $N_{gene}$ is the total number of genes. For cell type $C_i$, $N_i$ cells with the highest Gini coefficients are selected for poisoning.

Next, we perform poisoning operation on the selected cells. In detail, for each selected cell, we detect the genes with expression levels less than the poisoning threshold $\theta_p$ (default is two) and set the expression levels of these genes to zero. For the remaining genes, we randomize their expression levels and ensure that the total sum of all gene expression levels remains consistent before and after data poisoning, thus maintaining the sequencing depth constant. For example, for a cell with gene expression levels $\mathcal{E} = \{e_1, e_2, \ldots, e_{N_{gene}}\}$. Let $\mathcal{I} = \{i_1, i_2, \ldots, i_{N_{\theta_p}}\}$ indicate the indices of genes with expression levels higher than $\theta_p$, the changed expression levels $\mathcal{E}'$ of these genes are as follows:

$$\mathcal{E}' = \left\{e'_{i_1}, e'_{i_2}, \ldots, e'_{i_{N_{\theta_p}}}\right\}$$

$$e'_{i_j} = r_{i_j} \times \frac{\sum_{k=1}^{N_{gene}} e_k}{\sum_{j=1}^{N_{\theta_p}} r_{i_j}}, \qquad r_{i_j} \sim Uniform(0,1)$$

Finally, labels of the selected and poisoned cells are set as the target label.

**Supplementary Text S5. Experiments on scGPT and scBERT**

We conducted further experiments on the scGPT and scBERT using three different single-cell RNA sequencing (scRNA-seq) datasets. The three datasets are as follows:

1. The Myeloid dataset can be accessed from the GEO database using accession number GSE154763 (https://www.ncbi.nlm.nih.gov/geo/query/acc.cgi?acc=GSE154763)[33]. This dataset contains nine different cancer types. For model evaluation, we refer to the segmentation of this dataset in scGPT: six cancer types were selected for training, three cancer types were used for test.

2. The Gastric cancer dataset is available on the GEO database with accession number GSE206785 (https://www.ncbi.nlm.nih.gov/geo/query/acc.cgi?acc=GSE206785)[34]. We used the normal cells from this dataset for model evaluation.

3. The Heart dataset is a subset of the Tabula Sapiens, a benchmark human cell atlas of nearly 500,000 cells from 24 organs of 15 healthy individuals[35]. This dataset can be downloaded from https://figshare.com/articles/dataset/Tabula_Sapiens_release_1_0/14267219?file=34701976.

First, for the Myeloid dataset, since the cancer labels in the training and test sets do not overlap, we did not conduct a performance analysis on clean data in this case. We randomly selected one cancer type from the six types in the training set as the target label. The ASR remains high for both scGPT (0.986) and scBERT (0.987), indicating that the poisoned models can misclassify other types of cancer cells. Moreover, since the cancer labels between the training and test sets do not overlap, this shows that the poisoned model can still successfully attack unseen data. Extending this to real world scenarios, a poisoned model could misdiagnose cancer types and assign incorrect diagnoses, posing significant risks if maliciously exploited.

Next, as Table S1 shows, we selected two rare cell types in the Heart dataset as target labels (Cardiac Fibroblast-2.17%, Smooth Muscle Cells-1.93%) for poisoning attacks. The performance of the poisoned model on clean data is almost identical to that of the benign model, while the ASR remains high on poisoned data. The attack is both effective and covert, as the poisoned model can classify other cell as these rare types.

Finally, for the Gastric cancer dataset (Table S2), the performance of the poisoned model on

clean data is also very similar to that of the benign model, with a high ASR on poisoned data.

Overall, based on the above results, it can be observed that our backdoor strategy for scGPT and scBERT performs well on multiple datasets, ensuring good performance on clean data and maintaining high ASR.

**Supplementary Text S6. The impact of batch effects on the performance of backdoor attacks**

We conducted further experiments to explore whether batch effects influence the performance of backdoor attacks. We collected three scRNA-seq datasets with noticeable batch effects (Fig. S1), the specific information is as follow:

1. The Brain dataset is available on the GEO database with accession number GSE261157 (https://www.ncbi.nlm.nih.gov/geo/query/acc.cgi?acc=GSE261157). This dataset contains scRNA-seq profiles from 3D-cultured neural and cortical organoids derived from human iPS cells with a GFAP (R239C) mutation.

2. The Bone marrow and Tongue datasets are the subsets of the Tabula Sapiens, a benchmark human cell atlas of nearly 500,000 cells from 24 organs of 15 healthy individuals[35].

For each of the three datasets, we divided them into training and test sets, with the batch containing more cells designated as the training set and the batch containing less cells as the test set. Then we randomly selected a target label. Other parameter settings remain the same as before.

The results demonstrated that ASR exhibits slight fluctuations between different datasets, but overall remains at a relatively high level with 0.969 for the Brain dataset, 0.939 for the Bone marrow dataset, 0.941 for the Tongue dataset.

Furthermore, theoretically, the effectiveness of the attack should not be significantly impacted by the batch differences. This is because the essence of the attack lies in the model memorizing the trigger, which is a specific pattern. As long as this pattern appears, the attack will remain effective regardless of the batch in which it is encountered. In conclusion, these results demonstrated that our backdoor strategy is effectively robust to batch effects.

**Supplementary Text S7. The impact of feature selection on the performance of backdoor attacks**

We conducted the experiment about how feature selection influences the performance of backdoor attacks.

To simulate the scenario where different feature selection strategies are applied to the poisoned dataset during both the training and inference stages, we maintained the selection of the top 3,000 highly variable genes (HVGs) in the training set, while varying the number of highly variable genes selected in the test set, specifically at 2,750, 2,500, 2,250, 2,000, and 300, respectively. The reason for designing experiments in this way is to control the number of feature intersections in the training set and test set for quantitative evaluation.

As Table S3 shows, when the number of feature intersections (i.e., the number of feature intersection equals to the number of highly variable genes in the test set) in the training set and test set is within a certain range (i.e., number of highly variable genes in the test set is between 2,000 and 2,750), firstly, the performance of the poisoned model on the clean data remains good (Baseline: Accuracy = 0.968, Kappa = 0.954, and Macro-F1 = 0.710). Secondly, the attack effectiveness is barely affected. However, as the difference grows much larger (i.e., number of highly variable genes in the test set is 300), the attack effectiveness weakens, while the performance on clean data deteriorates. In conclusion, on one hand, feature selection does not enhance the effectiveness of backdoor attacks. On the other hand, it can’t serve as a defense mechanism against such attacks, because using different feature selection methods to significantly reduce ASR also leads to a significant drop in performance on clean data.

Overall, the results demonstrated that our backdoor strategy exhibits a certain degree of robustness when facing different feature selection strategies during both training and inference stages.

## Supplementary Text S8. The backdoor strategy for GeneFormer

GeneFormer differs from scGPT and scBERT as its model requires the rank value encoding of the transcriptome of each cell as inputs[12]. Consequently, the example dataset provided by GeneFormer does not include gene expression data but features a sorted list of gene IDs according to gene expression levels. To assess potential vulnerabilities of GeneFormer, we propose a tailored poisoning strategy for this dataset configuration. The strategy can also be divided into two stages:

Firstly, in the process of selecting cells from the non-target label cell types, we employed random sampling rather than using the Gini coefficient as a criterion, because we cannot calculate the Gini coefficient without gene expression levels.

Secondly, for each of the cells selected to be poisoned, we retain only the top $h$ (default is 100) gene IDs and shuffle their order randomly.

## Supplementary Text S9. Experiments on GeneFormer

We conducted further experiments on GeneFormer using three different scRNA-seq datasets. The three datasets are subsets of the example dataset of GeneFormer, which can be collected from https://huggingface.co/datasets/ctheodoris/Genecorpus-30M/tree/main/example_input_files/cell_classification/cell_type_annotation and can be divided into multiple subsets by different organs. Here, three subsets are selected for the experiment, namely Brain, Immune, and Spleen. For each of these three datasets, we randomly select the target label, the other settings remain the same as before.

As Table S4 shows, the performance of the poisoned model on clean data is similar to that of the benign model, while maintaining an ASR of 1.00. Our backdoor strategy for GeneFormer achieves strong performance across various datasets, delivering good results on clean data while consistently maintaining a high ASR. These results further demonstrated the effectiveness and robustness of our backdoor strategy.

## Supplementary Text S10. Details of the defense mechanisms

We provided implementation guidance for the five defense mechanisms, along with a discussion of their effectiveness, feasibility, and cost-effectiveness:

**Verifying the integrity of downloaded data or pretrained models.** Verifying the integrity of downloaded data or pretrained models is an effective method to prevent tampering. It works by checking the integrity of data or model files, effectively identifying any that have been compromised with. This is relatively easy to implement using technologies such as hash functions or digital signatures, with a low technical threshold. The cost is minimal, mainly incurred in computing hash values or verifying signatures, which is acceptable for large-scale data processing systems. The specific implementation involves data-provider using SHA-256[36] or other secure hash algorithms to compute hash value of the file and store the hash value on the server. After downloading the file, users should compute the hash value of the downloaded file locally, and then compare this newly computed hash value with the original hash value stored on the server. If these two hash values match, it indicates that the downloaded file has not been compromised with and can be safely used. Otherwise, it is unsafe, as it may suggest that the file has been poisoned, potentially due to attacks such as a man-in-the-middle attack. For research labs with limited resources, it is a simple but effective defense mechanism.

**Data inspection and sanitization.** These mechanisms, such as preprocessing-based defenses, have been proven effective in mitigating backdoor attacks by altering trigger patterns in input data. In the recent study, a pre-trained auto-encoder was utilized to modify trigger regions, thereby reducing ASR[37]. Another mechanism, known as Februus, employed GradCAM to detect influential regions and Generative Adversarial Network-based (GAN-based) inpainting to neutralize them while maintaining benign accuracy[38]. These mechanisms are feasible, as they integrate with existing pipelines. A lightweight trigger blocker was also proposed, using dominant colors to disrupt triggers at a low computational cost[39]. Overall, these mechanisms balance effectiveness, feasibility, and cost-efficiency well. However, designing such an effective mechanism requires a lot of experimentation and effort. Therefore, we hope that our work can inspire further research into these defense mechanisms within the single-cell field.

**Incorporating anomaly detection algorithms.** Incorporating anomaly detection algorithms helps identify abnormal patterns or potential anomalies in the data, aiding in preempting potential security threats. Selecting or developing appropriate anomaly detection algorithms that suit specific data characteristics requires high standards for algorithm selection and tuning[40]. Although this incurs a relatively high cost, especially during the development and tuning stages, it is justifiable for critical application areas. The implementation process includes selecting appropriate anomaly detection methods based on data characteristics (e.g., time series[41]), and may require continuous training and optimization of the model to adapt to data changes. However, similar to the second mechanism mentioned earlier, it may require some targeted design and the design may be difficult.

**Purifying suspicious models by retraining with benign samples.** Purifying suspicious models by retraining them with benign samples is a basic but effective mechanism in reducing the risk of model attacks. Actually, this defense mechanism[42] is used as a fundamental component in multiple backdoor defense mechanisms, such as Fine tuning (FP)[43] and Neural Attention Distillation (NAD)[44]. This mechanism usually has an unstable success rate, and the amount of data and computing resources required to successfully clean the backdoor is uncertain. In different situations, there may exist significant changes, so multiple attempts are needed. The implementation guide suggests selecting a certain amount of high-quality, diverse benign data for retraining, and monitoring the model performance (i.e., using metrics to evaluate) during this process. This mechanism could be effective if there are sufficient computational resources and benign data.

**Incorporating backdoor defenses into model design.** Incorporating backdoor defenses into model design can prevent the success of backdoor attacks to some extent by considering security factors right at the model design stage. Although this defense mechanism increases the initial design and development costs of the model, it is an effective preventive mechanism for maintaining long-term model security. This also requires special design, such as employing sub module that detect and mitigate backdoor triggers like Neural Cleanse[45], which reversely engineers potential triggers to identify hidden threats, or using techniques like STRIP[46], which perturbs inputs to monitor output consistency and detect backdoors. Another option is to integrate

optimization-based mechanisms such as TABOR[47], which inspects and restores neural networks by removing backdoor vulnerabilities. However, this mechanism is more complex and difficult to design than other defense mechanisms.

**Supplementary Text S11. Motivation and relevant examples**

We discussed the motivation and relevant examples (i.e., attacks that have already occurred in other fields), effectively demonstrating that backdoor attacks are indeed a legitimate concern.

To our best knowledge, in the single-cell field, this is the first paper to discuss backdoor attacks. To further illustrate our motivation, we divide it into three parts:

First, extending backdoor attacks into the biomedical field is an urgent task because similar attacks have already appeared in fields such as autonomous driving and facial recognition, posing significant threats. For instance, in autonomous driving, a poisoned system could misclassify a stop sign as a speed-limit sign, creating a serious safety hazard. A similar situation could arise in the biomedical field. Imagine a simple case: a cancer patient visiting a hospital for a diagnosis, their malignant cells are fed into a model compromised by a backdoor attack. The model could erroneously classify the cancer cells as normal cells or misclassify them as another type of cancer cells. Such a medical mistake would lead to significant harm and danger, highlighting the need for research into backdoor attacks in biomedicine.

Furthermore, while backdoor attack research has advanced considerably in other fields such as CV[6, 7] and NLP[8-10], it remains largely underexplored in the single-cell analysis field. This imbalance leaves single-cell pretrained models vulnerable to attack. Our research aims to raise awareness within the community, alert the community to this potential risk, and encourage more research into both attacks and defenses to enhance security in the single-cell field.

Finally, it is often one groundbreaking attack paper that starts the research in any field, as seen with BadNets[6] in CV. What's more, backdoor attacks have not received widespread attention in the emerging field of single-cell analysis, so addressing this problem is a pressing task.

In summary, the concern about data being easily poisoned is reasonable. Our work, as the first to discuss backdoor attacks in the single-cell field, seeks to bring attention to this critical issue and inspires further research in this direction.

# Supplementary Figure

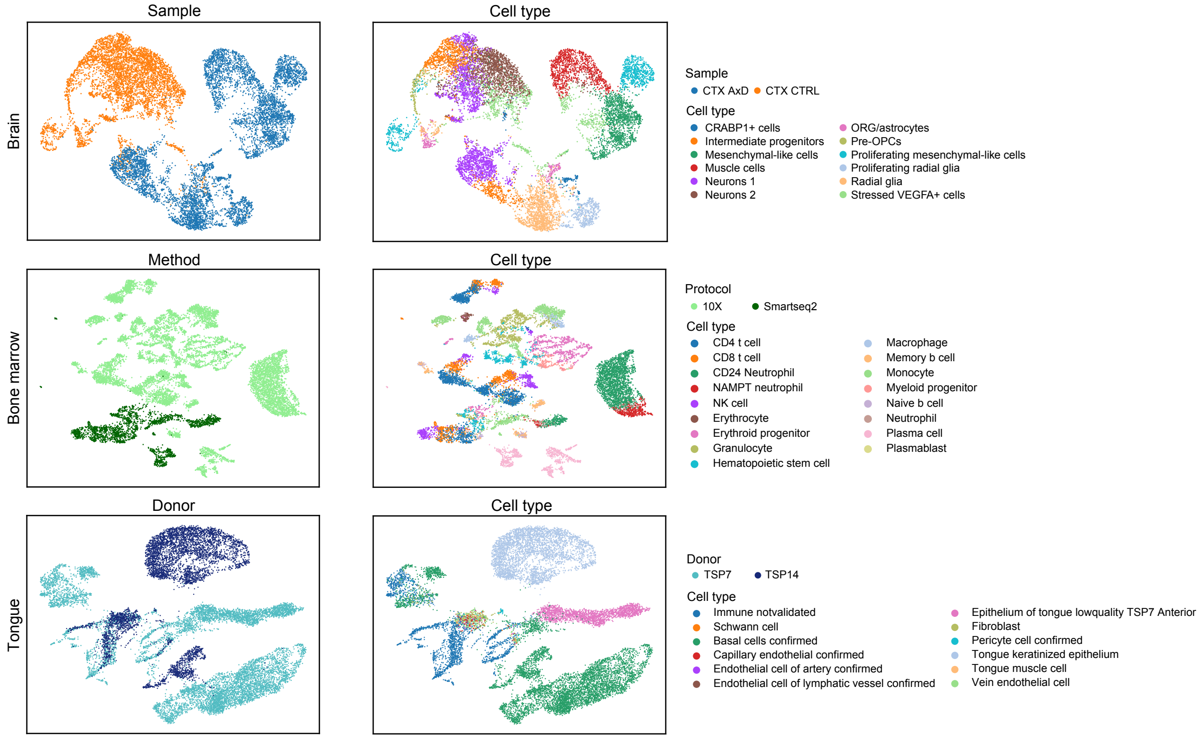


**Fig. S1 | UMAP visualization of three datasets with noticeable batch effects.** Cell type labels and batch labels (e.g., sample, protocol, and donor) are projected onto the visualizations, respectively.

## Supplementary Tables

**Table S1 | The performance of scGPT and scBERT on the Heart dataset.**

| | scGPT | | | scBERT | | |
|---|---|---|---|---|---|---|
| | Baseline | Cardiac Fibroblast | Smooth Muscle Cells | Baseline | Cardiac Fibroblast | Smooth Muscle Cells |
| Accuracy | 0.978 | 0.978 | 0.983 | 0.976 | 0.975 | 0.973 |
| Macro-F1 | 0.929 | 0.907 | 0.941 | 0.800 | 0.786 | 0.782 |
| Kappa | 0.959 | 0.960 | 0.970 | 0.954 | 0.954 | 0.950 |
| ASR | - | 0.970 | 0.957 | - | 0.931 | 0.920 |

**Table S2 | The performance of scGPT and scBERT on the Gastric cancer dataset.**

| | scGPT | | | scBERT | | |
|---|---|---|---|---|---|---|
| | Baseline | Innate lymphoid | Epithelial | Baseline | Innate lymphoid | Epithelial |
| Accuracy | 0.956 | 0.954 | 0.961 | 0.955 | 0.949 | 0.950 |
| Macro-F1 | 0.713 | 0.819 | 0.841 | 0.775 | 0.786 | 0.729 |
| Kappa | 0.928 | 0.926 | 0.937 | 0.925 | 0.916 | 0.917 |
| ASR | - | 0.958 | 0.981 | - | 0.890 | 0.888 |

**Table S3 | The performance of backdoor attacks at varying feature selections.** The clean data metrics and ASR of applying different feature selection to the poisoned dataset during both training and inference stages.

| Metrics | Number of HVGs selected in the test set = 2750 | Number of HVGs selected in the test set = 2500 | Number of HVGs selected in the test set = 2250 | Number of HVGs selected in the test set = 2000 | Number of HVGs selected in the test set = 300 |
|---|---|---|---|---|---|
| Accuracy | 0.962 | 0.967 | 0.958 | 0.975 | 0.707 |
| Macro-F1 | 0.661 | 0.735 | 0.734 | 0.759 | 0.449 |
| Kappa | 0.944 | 0.953 | 0.939 | 0.964 | 0.613 |
| ASR | 0.971 | 0.968 | 0.955 | 0.951 | 0.632 |

**Table S4 | The performance of GeneFormer on the Brain, Immune, and Spleen datasets.**

| | Brain | | Immune | | Spleen | |
|---|---|---|---|---|---|---|
| | Baseline | Fetal neuron | Baseline | Monocyte | Baseline | B cell (Plasmocyte) |
| Accuracy | 0.944 | 0.938 | 0.908 | 0.906 | 0.923 | 0.937 |
| Macro-F1 | 0.275 | 0.269 | 0.509 | 0.510 | 0.357 | 0.384 |
| Kappa | 0.761 | 0.718 | 0.872 | 0.868 | 0.822 | 0.855 |
| ASR | - | 1.000 | - | 1.000 | - | 1.000 |

## References


1. Yang, J., Wang, W. & Zhang, X. scSemiGCN: boosting cell-type annotation from noise-resistant graph neural networks with extremely limited supervision. *Bioinformatics* **40** (2024).

2. Kiselev, V.Y., Yiu, A. & Hemberg, M. Scmap: Projection of single-cell RNA-seq data across data sets. *Nat. Methods* **15**, 359-362 (2018).

3. Hu, J. et al. Iterative transfer learning with neural network for clustering and cell type classification in single-cell RNA-seq analysis. *Nat. Mach. Intell.* **2**, 607-618 (2020).

4. Janssen, P. et al. The effect of background noise and its removal on the analysis of single-cell expression data. *Genome Biol.* **24** (2023).

5. Cao, X. et al. scPriorGraph: constructing biosemantic cell–cell graphs with prior gene set selection for cell type identification from scRNA-seq data. *Genome Biol.* **25** (2024).

6. Gu, T., Dolan-Gavitt, B. & Garg, S. Badnets: Identifying vulnerabilities in the machine learning model supply chain. *arXiv preprint arXiv:1708.06733* (2017).

7. Chen, X., Liu, C., Li, B., Lu, K. & Song, D. Targeted backdoor attacks on deep learning systems using data poisoning. *arXiv preprint arXiv:1712.05526* (2017).

8. Chen, X. et al. BadNL: Backdoor Attacks against NLP Models with Semantic-preserving Improvements. *ACM International Conference Proceeding Series*, 554-569 (2021).

9. Pan, X., Zhang, M., Sheng, B., Zhu, J. & Yang, M. Hidden Trigger Backdoor Attack on NLP Models via Linguistic Style Manipulation. *Proceedings of the 31st USENIX Security Symposium, Security 2022*, 3611-3628 (2022).

10. Omar, M. Backdoor learning for nlp: Recent advances, challenges, and future research directions. *arXiv preprint arXiv:2302.06801* (2023).

11. Yang, F. et al. scBERT as a large-scale pretrained deep language model for cell type annotation of single-cell RNA-seq data. *Nat. Mach. Intell.* **4**, 852-866 (2022).

12. Theodoris, C.V. et al. Transfer learning enables predictions in network biology. *Nature* **618**, 616-624 (2023).

13. Cui, H. et al. scGPT: toward building a foundation model for single-cell multi-omics using generative AI. *Nat. Methods* (2024).

14. Biology, C.S.-C. et al. CZ CELLxGENE Discover: A single-cell data platform for scalable exploration, analysis and modeling of aggregated data. *BioRxiv*, 2023.2010. 2030.563174 (2023).

15. Barrett, T. et al. NCBI GEO: archive for functional genomics data sets—update. *Nucleic Acids Res.* **41**, D991-D995 (2012).

16. Frank, M., Ni, P., Jensen, M. & Gerstein, M.B. Leveraging a large language model to predict protein phase transition: A physical, multiscale, and interpretable approach. *Proc. Natl. Acad. Sci. U. S. A.* **121** (2024).

17. Elnaggar, A. et al. ProtTrans: Toward Understanding the Language of Life Through Self-Supervised Learning. *IEEE Transactions on Pattern Analysis and Machine Intelligence* **44**, 7112-7127 (2022).

18. Rives, A. et al. Biological structure and function emerge from scaling unsupervised learning to 250 million protein sequences. *Proc. Natl. Acad. Sci. U. S. A.* **118** (2021).

19. Ferruz, N., Schmidt, S. & Höcker, B. ProtGPT2 is a deep unsupervised language model for protein design. *Nat. Commun.* **13** (2022).

20. Izacard, G., Mohan, S. & Fernandez-Granda, C. Data-driven estimation of sinusoid frequencies. *Advances in Neural Information Processing Systems* **32** (2019).

21. Burley, S.K. et al. Protein Data Bank (PDB): the single global macromolecular structure archive. *Protein crystallography: methods and protocols*, 627-641 (2017).

22. Suzek, B.E., Huang, H., McGarvey, P., Mazumder, R. & Wu, C.H. UniRef: Comprehensive and non-redundant UniProt reference clusters. *Bioinformatics* **23**, 1282-1288 (2007).

23. Suzek, B.E., Wang, Y., Huang, H., McGarvey, P.B. & Wu, C.H. UniRef clusters: A comprehensive and scalable alternative for improving sequence similarity searches. *Bioinformatics* **31**, 926-932 (2015).

24. Richardson, L. et al. MGnify: the microbiome sequence data analysis resource in 2023. *Nucleic Acids Res.* **51**, D753-D759 (2023).

25. Sanabria, M., Hirsch, J., Joubert, P.M. & Poetsch, A.R. DNA language model GROVER learns sequence context in the human genome. *Nat. Mach. Intell.* **6**, 911-923 (2024).

26. Benegas, G., Batra, S.S. & Song, Y.S. DNA language models are powerful predictors of genome-wide variant effects. *Proc. Natl. Acad. Sci. U. S. A.* **120** (2023).

27. Nguyen, E. et al. Hyenadna: Long-range genomic sequence modeling at single nucleotide resolution. *Advances in neural information processing systems* **36** (2024).

28. Schoch, C.L. et al. NCBI Taxonomy: a comprehensive update on curation, resources and tools. *Database* **2020**, baaa062 (2020).

29. Chen, X. et al. Cell type annotation of single-cell chromatin accessibility data via supervised Bayesian embedding. *Nat. Mach. Intell.* **4**, 116-126 (2022).

30. Ma, W., Su, K. & Wu, H. Evaluation of some aspects in supervised cell type identification for single-cell RNA-seq: classifier, feature selection, and reference construction. *Genome Biol.* **22**, 1-23 (2021).

31. Li, Y., Jiang, Y., Li, Z. & Xia, S.T. Backdoor learning: a survey. *IEEE Trans. Neural Netw. Learn. Syst.* **35**, 5-22 (2024).

32. Jiang, Y., Ma, X., Erfani, S.M. & Bailey, J. Backdoor attacks on time series: A generative approach. *2023 IEEE Conference on Secure and Trustworthy Machine Learning (SaTML)*, 392-403 (2023).

33. Cheng, S. et al. A pan-cancer single-cell transcriptional atlas of tumor infiltrating myeloid cells. *Cell* **184**, 792-809.e723 (2021).

34. Kang, B. et al. Parallel single-cell and bulk transcriptome analyses reveal key features of the gastric tumor microenvironment. *Genome Biol.* **23** (2022).

35. Jones, R.C. et al. The Tabula Sapiens: A multiple-organ, single-cell transcriptomic atlas of humans. *Science* **376** (2022).

36. Standard, D.E. Federal information processing standards publication 46. *National Bureau of Standards, US Department of Commerce* **23**, 1-18 (1977).

37. Liu, Y., Xie, Y. & Srivastava, A. Neural trojans. *Proceedings - 35th IEEE International Conference on Computer Design, ICCD 2017*, 45-48 (2017).

38. Doan, B.G., Abbasnejad, E. & Ranasinghe, D.C. Februus: Input Purification Defense against Trojan Attacks on Deep Neural Network Systems. *ACM International Conference Proceeding Series*, 897-912 (2020).

39. Udeshi, S. et al. Model Agnostic Defence Against Backdoor Attacks in Machine Learning. *IEEE Transactions on Reliability* **71**, 880-895 (2022).

40. Qiu, C., Li, A., Kloft, M., Rudolph, M. & Mandt, S. Latent Outlier Exposure for Anomaly Detection with Contaminated Data. *Proceedings of Machine Learning Research* **162**, 18153-18167 (2022).

41. Rebjock, Q., Kurt, B., Januschowski, T. & Callot, L. Online false discovery rate control for anomaly detection in time series. *Advances in Neural Information Processing Systems* **32**, 26487-26498 (2021).

42. Wu, B. et al. Backdoorbench: A comprehensive benchmark of backdoor learning. *Advances in Neural Information Processing Systems* **35**, 10546-10559 (2022).

43. Liu, K., Dolan-Gavitt, B. & Garg, S. Fine-pruning: Defending against backdooring attacks on deep neural networks. *International symposium on research in attacks, intrusions, and defenses*, 273-294 (2018).

44. Li, Y. et al. Neural attention distillation: Erasing backdoor triggers from deep neural networks. *arXiv preprint arXiv:2101.05930* (2021).

45. Wang, B. et al. Neural cleanse: Identifying and mitigating backdoor attacks in neural networks. *2019 IEEE Symposium on Security and Privacy (SP)*, 707-723 (2019).

46. Gao, Y. et al. Strip: A defence against trojan attacks on deep neural networks. *Proceedings of the 35th annual computer security applications conference*, 113-125 (2019).

47. Guo, W., Wang, L., Xing, X., Du, M. & Song, D. Tabor: A highly accurate approach to inspecting and restoring trojan backdoors in ai systems. *arXiv preprint arXiv:1908.01763* (2019).
